# Primary tumor site is a useful predictor of cetuximab efficacy in the third-line or salvage treatment of *KRAS* wild-type (exon 2 non-mutant) metastatic colorectal cancer: a nationwide cohort study

**DOI:** 10.1186/s12885-016-2358-2

**Published:** 2016-05-24

**Authors:** Kuo-Hsing Chen, Yu-Yun Shao, Ho-Min Chen, Yu-Lin Lin, Zhong-Zhe Lin, Mei-Shu Lai, Ann-Lii Cheng, Kun-Huei Yeh

**Affiliations:** Department of Oncology, National Taiwan University Hospital, 7, Chun-Shan S Rd, Taipei, 10002 Taiwan; Center for Comparative Effectiveness Research, National Center of Excellence for Clinical Trial and Research, National Taiwan University Hospital, Taipei, Taiwan; National Taiwan University Cancer Center, Taipei, Taiwan; Graduate Institute of Oncology, College of Medicine, National Taiwan University, Taipei, Taiwan; Graduate Institute of Clinical Medicine, College of Medicine, National Taiwan University, Taipei, Taiwan; Department of Internal Medicine, College of Medicine, National Taiwan University, Taipei, Taiwan; Taiwan Cancer Registry, Taipei, Taiwan; Graduate Institute of Epidemiology and Preventive Medicine, College of Public Health, National Taiwan University, Taipei, Taiwan

**Keywords:** Cetuximab, Colorectal cancer, Primary tumor site, Predictive biomarker, *KRAS* wild-type

## Abstract

**Background:**

Previous studies have shown left-sided colorectal cancer (LCRC) and right-sided colorectal cancer (RCRC) exhibit different molecular and clinicopathological features. We explored the association between the primary tumor site and cetuximab efficacy in *KRAS* wild-type colorectal cancer (CRC).

**Methods:**

This study enrolled a cohort of patients, who had received cetuximab treatment after two or more lines of chemotherapy for *KRAS* wild-type (exon 2 nonmutant) metastatic CRC, from the databases of Taiwan Cancer Registry (2004–2010) and National Health Insurance (2004–2011). Survival data were obtained from the National Death Registry. Time to treatment discontinuation (TTD) and overall survival (OS) after the start of cetuximab treatment were compared between patients with LCRC (splenic flexure to rectum) and RCRC (cecum to hepatic flexure).

**Results:**

A total of 969 CRC patients were enrolled. Among them, 765 (78.9 %) and 136 (14.0 %) patients had LCRC and RCRC, respectively. Patients with LCRC, compared to patients with RCRC, had longer TTD (median, 4.59 vs. 2.75 months, *P* = .0005) and OS (median, 12.62 vs. 8.07 months, *P* < .0001) after the start of cetuximab treatment. Multivariate analysis revealed a right-sided primary tumor site was an independent predictor of shorter TTD (adjusted hazard ratio [HR] = 1.32, using the LCRC group as a reference, 95 % confidence interval: 1.08–1.61, *P* = .0072) and OS (adjusted HR = 1.45, 95 % CI: 1.18–1.78, *P* = .0003).

**Conclusion:**

Our findings demonstrate that a left-sided primary tumor site is a useful predictor of improved cetuximab efficacy in the third-line or salvage treatment of *KRAS* wild-type (exon 2 nonmutant) metastatic CRC.

## Background

Monoclonal antibodies targeting the epidermal growth factor receptor (EGFR), either used alone, or a combination with cytotoxic agents have been demonstrated to prolong survival in patients with metastatic colorectal cancer (CRC) harboring *KRAS* wild-type or expanded *RAS* [1–6]. However, not all patients experienced clinical benefits of the anti-EGFR antibody treatment. These studies have emphasized the importance of additional predictive biomarkers for anti-EGFR antibody treatment. Some predictive biomarkers, such as the gene expression of EGFR ligands, have been reported to correlate with patient responses after the anti-EGFR antibody treatment [7, 8]. Besides, the primary resistance mechanisms of cetuximab have been investigated rigorously. The negative predictive roles of expanded *RAS* [*KRAS* (exons 2, 3, and 4) and *NRAS* (exons 2, 3, and 4)] have been well established, but other biomarkers, including *BRAF*^V600E^ mutation, amplification of *KRAS*, *MET*, and *ERBB2*, and cross-talk with PI3K/Akt/PETN,, still remain to be investigational [5, 6, 9–17].

Left- and right-sided CRC (LCRC and RCRC) have different clinicopathological and molecular characteristics [18–20]. Recently, clinical studies have shown that a left-sided primary tumor site was associated with the benefits of cetuximab, which is one of the anti-EGFR antibodies, in patients with *KRAS* wild-type (exon 2 nonmutant) CRC [21, 22]. A subgroup analysis of the AIO KRK-0306 trial revealed similar findings in patients with expanded *RAS* wild-type CRC [23]. The definitive reasons for this phenomenon remain unknown. Because most of the aforementioned studies have investigated Western populations, whether there is a similar association between a primary tumor site and cetuximab efficacy in the Taiwanese population has yet to be determined.

In Taiwan, patients have been reimbursed by the National Health Insurance (NHI) for cetuximab administration as the third-line or salvage therapy for *KRAS* wild-type (exon 2 nonmutant) metastatic CRC since August 1, 2009 [24]. In this study, we used the Taiwan Cancer Registry (TCR) and NHI databases concomitantly to evaluate the association between a primary tumor site and the clinical benefits of cetuximab in patients with *KRAS* wild-type (exon 2 nonmutant) metastatic CRC.

## Methods

### Data source

The TCR database, which is organized and funded by the Ministry of Health and Welfare, Taiwan, was implemented in 1979, and an excellent coverage rate (97 %) and data quality of cancer registry have been achieved [25]. Hospitals were enlisted to report information on all newly diagnosed cancers to the central registry office if they had 50 or more inpatient beds. For monitoring the patterns of cancer care and evaluates the outcomes of cancer treatment, the central cancer registry (a long-form database) has been modified since 2002 to include detailed items of the stage at diagnosis and the first course of treatment. Eighty hospitals, which account for more than 90 % of total cancer cases in Taiwan, are involved in the long-form registration.

NHI is a mandatory health insurance system, which covers more than 99 % of Taiwan’s population. The NHI database can provide patient medical records about diagnosis, clinical visits, admission, and drug prescriptions, and the claims data are representative nationally. The database has been developed as a tool for clinical cancer research [26] and was used in our study to collect complete records of the prescriptions of chemotherapy and cetuximab. The NHI claims data on every patient were examined thoroughly to determine the time of initiation and discontinuation of cetuximab and subsequent chemotherapy.

The medical records were also linked to the National Death Registry database to obtain mortality data and were traced until December 31, 2012. Personal identities were encrypted, and all data were analyzed anonymously to comply with privacy regulations. The study data were released after approval by the Data Release Review Boards of the Health Promotion Administration and Collaboration Center of Health Information Application, Ministry of Health and Welfare, Executive Yuan, Taiwan. The study protocol was approved by the Research Ethics Committee of National Taiwan University Hospital.

### Study population

A cohort of patients with a newly diagnosed CRC (ICD-O-3: C180–C189, C199, C209, excluding morphology codes representing lymphoma of 9590–9989 and Kaposi sarcoma of 9140) from 2004 to 2010 was identified from the TCR database. Patients were included in this study if they met the following criteria: [1] pathologically proven single primary CRC; [2] aged ≥ 18 years; [3] having known the cancer stage at diagnosis, according to the American Joint Committee on Cancer system, Sixth Edition; [4] having received standard chemotherapy (oxaliplatin, irinotecan, and one of the following: capecitabine, uracil–tegafur, or fluorouracil); and [5] having received more than one prescription of cetuximab as the third-line or salvage treatment for metastatic CRC and the first prescription of cetuximab during August 1, 2009 to December 31, 2011.

In Taiwan, since August 1, 2009, cetuximab treatment services have been reimbursed by the NHI for patients with *KRAS* wild-type (exon 2 nonmutant) metastatic CRC who failed to respond to oxaliplatin, irinotecan, and fluorouracil. Capecitabine and uracil–tegafur are commonly recognized alternatives to fluorouracil; thus, some patients who used capecitabine or uracil–tegafur instead of fluorouracil were also reimbursed. Physicians had to provide documentation of pathology, images, prior chemotherapy records, and *KRAS* mutation tests when applying to the NHI for cetuximab reimbursement. The amount of cetuximab for which reimbursement would be provided at a given time was a standard dosage (250 mg/m^2^ per week) for 9 weeks. The physicians were mandated to submit image reports supporting the presence of a responsive or stable disease after cetuximab treatment to apply for the second round of 9-week cetuximab usage. The maximal amount of reimbursed cetuximab treatment by the NHI was the standard dosage for 18 weeks.

### Study variables and outcomes

The baseline characteristics of the study patients, including age (grouped as < 50 years, 50–64 years, and > 65 years), sex, histology, the cancer stage at diagnosis, cancer grading, and primary tumor site were retrieved from the TCR database. Patients were classified into either an RCRC or LCRC group, where RCRC was defined as cancer from the cecum to hepatic flexure of the colon (ICD-O-3: C180–C183), and LCRC (ICD-O-3: C185, 186, 187, 199, 209) was defined as cancer from the splenic flexure of the colon to the rectum.

The main endpoints were overall survival (OS) and time to treatment discontinuation (TTD). OS was determined from the initiation of cetuximab treatment to the time of death or until December 31, 2012, whichever came first. TTD was calculated from the initiation of cetuximab to the date of the final cetuximab prescription, the date of death, or December 31, 2012, whichever came first.

### Statistical analysis

The mean demographic and clinical characteristics of patients with LCRC and RCRC at baseline were compared using the chi-squared test for categorical variables and the two-sample *t* test for continuous variables. OS and TTD were estimated using the Kaplan–Meier method, and comparisons were made using the log-rank test. The Cox proportional hazard model was used to estimate adjusted hazard ratios (HRs) and 95 % confidence intervals (CIs). The age, sex, histology, cancer stage at diagnosis, and tumor grade of the patients were adjusted using the Cox proportional hazard model. For comparison, results with a two-sided *P* value of less than .05 were considered statistically significant. Statistical software, SAS (Version 9.3, SAS Institute, Cary, NC, USA), was used for all statistical analyses.

## Results

**Fig. 1:**
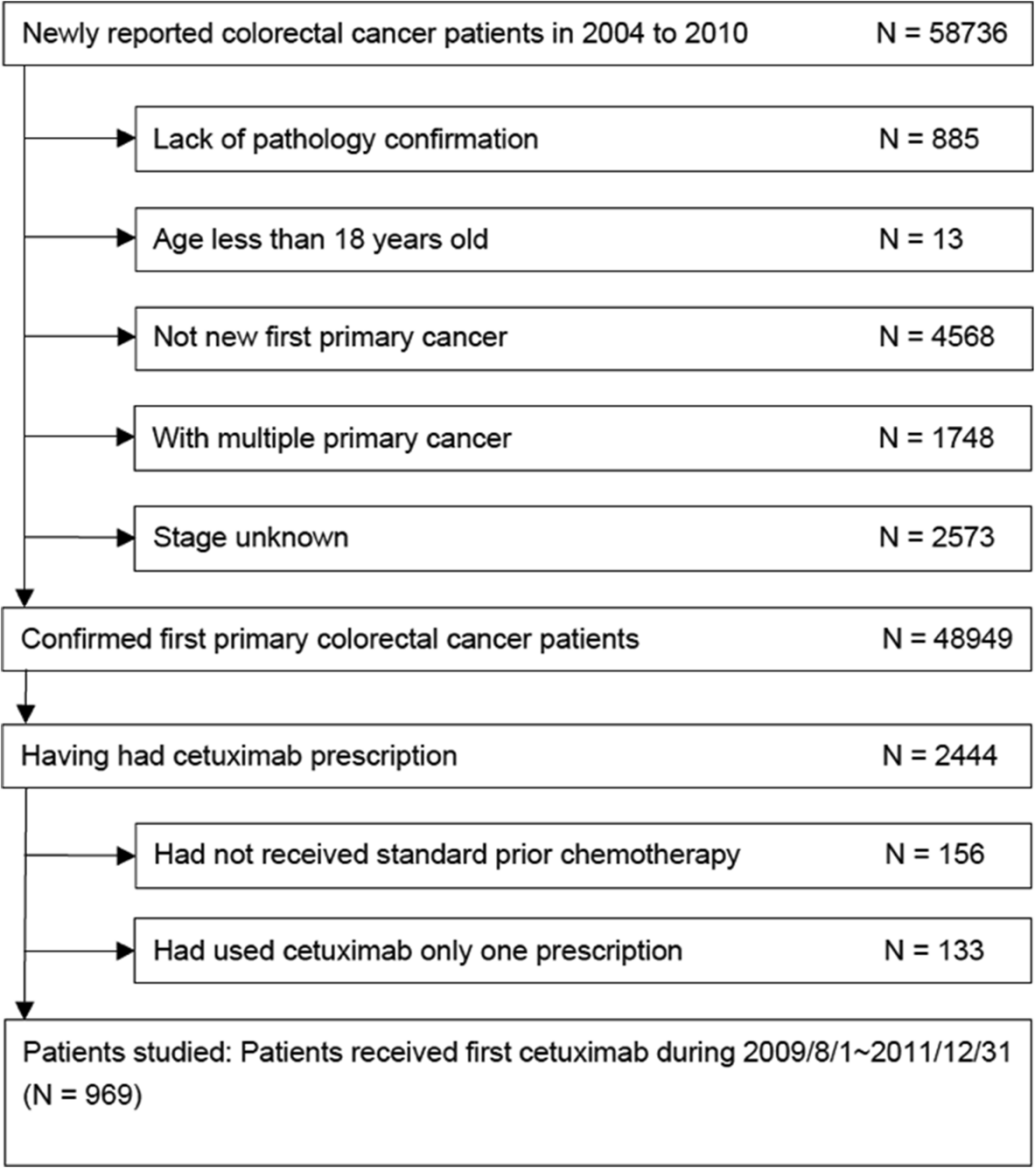
Consort diagram illustrating the treatment flow of patients

A total of 58 736 patients with a newly diagnosed CRC were identified from the TCR database; among them, 969 patients met the inclusion criteria and were enrolled in this study (Fig. 1). The study population comprised 591 (61 %) males with a median age at cetuximab treatment of 60 years, 938 (96.8 %) patients with adenocarcinoma, and 136 (14 %), 58 (6 %), and 765 (78.9 %) patients with right-sided, transverse, and left-sided primary tumor sites, respectively (Table 1). Five hundred and fifty (56.8 %) patients had initial stage IV CRC. The mean time interval from diagnosis to the first cetuximab prescription was 26.4 months. Nearly all (99.2 %) patients received cetuximab treatment in combination with chemotherapy.

**Table 1:** Patient characteristics of all studied patients

	N (%)
Patient Number	969 (100.0)
Gender	
Male	591 (61.0)
Female	378 (39.0)
Age at treatment (years)	
Mean (SD)	60.01 (12.11)
Median (min, max)	60 (22, 96)
Side	
Left (splenic flexture to rectum)	765 (78.9)
Right (cecum to hepatic flexture)	136 (14.0)
Transverse	58 (6.0)
Unknown	10 (1.0)
Histology	
Adenocarcinoma	938 (96.8)
Others	31 (3.2)
Grade	
1	46 (4.7)
2	738 (76.2)
3	103 (10.6)
Undifferentiated	6 (0.6)
Unknown	76 (7.8)
Stage at diagnosis^a^	
I-III	419 (43.2)
IV	550 (56.8)
Time interval from diagnosis date to first prescription of Cetuximab	
Mean months (SD)	26.4 (15.3)
Cetuximab combination with chemotherapy	961 (99.2)
Chemotherapy after end of Cetuximab	532 (54.9 %)
Death	806 (83.2)
Follow-up (months)	
Mean (SD)	12.8 (8.6)
Median (min, max)	11.3 (0.1, 39.4)

Abbreviation: *SD* standard deviation

^a^by American Joint Cancer Committee on Cancer (AJCC) system, 6^th^ edition

**Fig. 2:**
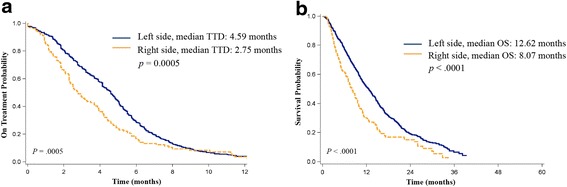
Kaplan-Meier analysis of time to treatment discontinuation and overall survival. Kaplan-Meier analysis of time to treatment discontinuation (**a**) and overall survival (**b**) among patients who received cetuximab as salvage therapy for advanced KRAS wild type (exon 2 non-mutant) CRC. Patients were divided according to primary tumor site (left side: splenic flexture to rectum; right side: cecum to hepatic flexture). The *P* values were conducted using the log-rank test

Patients with a primary site of cancer at the transverse colon or an unspecified site were excluded from survival analysis. Compared with patients with LCRC, patients with RCRC were mostly female (45.6 % vs. 36.9 %, *P* = .0536) and showed more mucinous adenocarcinoma (11 % vs. 2.7 %, *P* < .0001) and grade 3 tumors (20.6 % vs. 8.5 %, *P* = .002) (Table 2). The median follow-up time was 11.5 months. Patients with LCRC had significantly longer OS (median, 12.62 vs. 8.07 months, *P* < .0001) and TTD (median, 4.59 vs. 2.75 months, *P* = .0005) than those of patients with RCRC (Fig. 2). After we adjusted the covariates, including age at cetuximab treatment, sex, histology, stage at diagnosis, and tumor grade, RCRC was an independent predictor of overall mortality (adjusted HR = 1.45 [using the LCRC group as a reference], 95 % confidence of interval (CI): 1.18–1.78, *P* = .0003) after cetuximab treatment and treatment discontinuation (adjusted HR = 1.32, 95 % CI: 1.08–1.61, *P* = .0072) (Table 3).

**Table 2:** Patient characteristics of LCRC and RCRC

	Total	RCRC	LCRC	
	N (%)	N (%)	N (%)	*P* value
Patient Number	901 (100.0)	136 (100.0)	765 (100.0)	
Gender				
Male	557 (61.8)	74 (54.4)	483 (63.1)	.0536
Female	344 (38.2)	62 (45.6)	282 (36.9)	
Mean age at treatment (years)				
Mean (SD)	59.95 (12.02)	61.39 (11.91)	59.70 (12.02)	.0992
Median (min, max)	60 (22, 96)	61 (22, 96)	60 (26, 90)	
Age group (years)				
<50	163 (18.1)	18 (13.2)	145 (19.0)	.2256
50-64	420 (46.6)	64 (47.1)	356 (46.5)	
65+	318 (35.3)	54 (39.7)	264 (34.5)	
Histology				
Mucinous adenocarcinoma	36 (4.0)	15 (11.0)	21 (2.7)	< .0001
Non-mucinous adenocarcinoma	836 (92.8)	115 (84.6)	721 (94.2)	
Others	29 (3.2)	6 (4.4)	23 (3.0)	
Grade				
1	43 (4.8)	5 (3.7)	38 (5.0)	.0002
2	689 (76.5)	90 (66.2)	599 (78.3)	
3	93 (10.3)	28 (20.6)	65 (8.5)	
Undifferentiated or Unknown	76 (8.4)	13 (9.6)	63 (8.2)	
Stage at diagnosis^a^				
I-III	396 (44.0)	64 (47.1)	332 (43.4)	.4281
IV	505 (56.0)	72 (52.9)	433 (56.6)	
Cetuximab combination with chemotherapy	893 (99.1)	135 (99.3)	758 (99.1)	
Chemotherapy after end of cetuximab	495 (54.9)	61 (44.9)	434 (56.7)	
Death	747 (82.9)	121 (89.0)	626 (81.8)	
Follow-up (months)				
Mean (SD)	13.0 (8.6)	10.1 (8.0)	13.5 (8.6)	
Median (min, max)	11.5 (0.1, 39.4)	8.1 (1.2, 34.8)	12.5 (0.1, 39.4)	

Abbreviation: *SD* standard deviation

^a^by American Joint Cancer Committee on Cancer (AJCC) system, 6^th^ edition

**Table 3:** Multivariate analyses of overall mortality and treatment discontinuation. Multivariate analysis by a Cox’s proportional hazard model for hazard ratios of overall mortality and treatment discontinuation in patients received cetuximab as salvage treatment for advanced KRAS wild type (exon 2 non-mutant) CRC

	Overall mortality		Treatment discontinuation	
Variable	Adjusted HR (95 % CI)	*P* value	Adjusted HR (95 % CI)	*P* value
RCRC (vs. LCRC)	1.45 (1.18, 1.78)	.0003	1.32 (1.08, 1.61)	.0072
Female (vs. male)	1.04(0.90, 1.21)	.5869	1.01 (0.87, 1.16)	.9413
Age (vs. < 50 y)		.2962		.0783
50-64	0.86 (0.70, 1.06)		0.80 (0.66, 0.98)	
65+	0.94 (0.76, 1.17)		0.82 (0.67, 1.01)	
Stage IV at diagnosis (vs. I-III)^a^	1.11 (0.95, 1.28)	.1887	1.08 (0.94, 1.25)	.2916
Histology (vs. others)		.1096		.1524
Mucinous adenocarcinoma	1.55 (0.91, 2.65)		1.69 (0.99, 2.89)	
Non-mucinous adenocarcinoma	1.04 (0.69, 1.59)		1.42 (0.93, 2.18)	
Grade (vs. I)		.0009		.0023
II	1.24 (0.88, 1.76)		1.02 (0.74, 1.40)	
III	1.82 (1.21, 2.74)		1.39 (0.95, 2.05)	
Undifferentiated or unknown	1.75 (1.15, 2.65)		1.50 (1.01, 2.22)	

Abbreviation: *HR* hazard ratio, *CI* confidence interval, *RCRC* right sided colorectal cancer (cecum to hepatic flexture), *LCRC* left sided colorectal cancer (splenic flexture to rectum), *SD* standard deviation

^a^by American Joint Cancer Committee on Cancer (AJCC) system, 6^th^ edition

## Discussion

In this study, we found that patients with LCRC had more clinical benefits of the third-line or salvage cetuximab treatment regarding TTD and OS than did patients with RCRC. In addition, multivariate analysis demonstrated that a primary tumor site was an independent predictor of patient prognosis. TTD, instead of traditional progression-free survival (PFS), was used as one of the endpoints of the study because the maximal reimbursement amount of cetuximab was the standard 18-week dosage and the time of disease progression in patients were not recorded in NHI and TCR databases. Our study was in agreement with several studies on Western population. In an exploratory analysis of NCIC CTG CO.17, Brulé et al demonstrated that a left-sided tumor site (splenic flexure to rectosigmoid colon) is a strong predictive factor for long PFS in patients with refractory, metastatic, and *KRAS* wild-type (exon 2) colon cancer receiving cetuximab treatment [27]. Two AIO KRK studies (0104 and 0306) demonstrated that in populations with either *KRAS* wild-type (codon 12/13) or expanded *RAS*, a left primary tumor site was associated with long PFS and OS in untreated metastatic CRC patients who received cetuximab-containing regimens [21, 23]. However, based on our research, the current study is the first to demonstrate an association between the primary tumor site of CRC and the clinical benefits of cetuximab treatment in the East Asian population.

The definitive reasons for different clinical benefits of cetuximab treatment in these patients remain unclear. Several studies have revealed different molecular and clinicopathological features between left-sided and right-sided CRC [18–20, 28]. For example, RCRC is characterized by features such as microsatellite instability phenotype, *RAS* mutation, mitogen-activated protein kinase activation, *BRAF*^*V600E*^ mutation, *BRAF*-like characteristics, and the CpG island methylator phenotype [18, 20]. By contrast, LCRC is characterized by chromosomal instability, amplification of *EGFR* and *ERBB2*, EGFR pathway upregulation, and WNT, MYC, and SRC pathway activation [18]. Because the aforementioned association of a primary tumor site with cetuximab treatment was also noted in an expanded *RAS* wild-type population, primary expanded *KRAS* (exon 2, 3, 4) or *NRAS* (exon 2, 3, 4) mutations may not explain the different clinical benefits of cetuximab treatment in patients with different primary tumor sites. Whether emergence of new *KRAS* mutations played a role is unknown. Several studies have suggested that the high gene expression of EGFR ligands (epiregulin and amphiregulin) predicted favorable outcomes in patients receiving cetuximab treatment, which may explain our findings [7, 8]. *BRAF*^*V600E*^ has been shown to have a strong causal relationship with resistance to anti-EGFR antibodies in preclinical models, but the correlation in clinical settings is not statistically significant [9, 12, 13, 17]. There are several gene alterations involved in the EGFR signaling pathway beyond RAS and BRAF mutations, which converge biochemically on activation of RAS/MEK/ERK, but their relevance to the de novo resistance to anti-EGFR antibody treatment remains undetermined.

Although many studies have shown promising efficacies of anti-EGFR antibody therapy in *KRAS* or expanded *RAS* wild-type metastatic CRC, the heterogeneity of CRC should be considered if different benefits were found in patient subsets. Our study showed a poor cetuximab treatment efficacy in patients with *KRAS* wild-type RCRC (cecum to hepatic flexure), thus emphasizing an urgent unmet clinical need in such patients. Bevacizumab is a vascular endothelial growth factor inhibitor that has shown clinical benefits in patients with advanced CRC [6, 29]. In the post hoc analysis of an AIO KRK-0306 trial, patients with RCRC appeared to have a favorable outcome in the bevacizumab plus FOLFIRI arm [23]. Whether bevacizumab is a superior choice for patients with *RAS* wild-type RCRC must be validated in a randomized clinical trial. Recently, a combination of an anti-EGFR antibody and an MEK inhibitor has been expected to overcome the resistance emerging from *KRAS* mutations or cross talk with the PI3K/Akt/PETN pathway after anti-EGFR antibody treatment, and the upfront use of these regimens could be considered in these patient subsets [30].

There are limitations of the current study. First, this was a nationwide cohort study, and we identified patients with *KRAS* wild-type CRC according to their usage of reimbursed cetuximab. Thus, we could not perform expanded *KRAS* or *NRAS* analyses during the study period. Moreover, the schedule and dosage of cetuximab could not be uniform, which might have confounded our results, particularly for TTD. Second, bevacizumab was not reimbursed for advanced CRC until June 1, 2011 (in a first-line setting only) but was approved by the Taiwan Food and Drug Administration in 2005. Previous studies have shown that patients with different primary tumor sites might derive different benefits from bevacizumab combined with chemotherapy [23, 31]. A few patients who used self-paid bevacizumab might have confounded our results. Third, some experts have postulated that molecular features of CRC change gradually with the bowel; thus, it might be oversimplified to classify heterogeneous CRC as only a left- or right-sided group [32]. Moreover, although Brulé et al revealed primary tumor site was not a prognostic factor in refractory CRC patients in NCIC CO.17, the prognostic role of it in *KRAS* or *RAS* wild type, metastatic CRC patients remains unknown [22]. We could not exclude the probability that left-sided tumor was a favorable prognostic factor. The long interval between TTD and OS may also imply other confounding factors were ignored. However, our study clearly demonstrated that the primary tumor site (left- or right-sided) is a useful biomarker for predicting the prognosis after cetuximab treatment in patients with advanced *KRAS* wild-type (exon 2 nonmutant) CRC. Furthermore, our nationwide study had the advantages of evaluating OS and preventing selection bias, because no eligible patients were lost to follow-up.

## Conclusion

This study demonstrated that a left-sided primary tumor site is a useful predictive marker for improved cetuximab efficacy for the third-line or salvage treatment among patients with *KRAS* wild-type (exon 2 nonmutant) metastatic CRC. Our study results emphasize the unmet medical needs in patients with a right-sided tumor site and provide factual survival data for future clinical trials.

## Abbreviations

AJCC: American Joint Committee on Cancer
CI: confidence intervals
CRC: colorectal cancer
EGFR: epidermal growth factor receptor
HR: hazard ratio
LCRC: left-sided colorectal cancer
NHI: National Health Insurance
OS: overall survival
PFS: progression-free survival
RCRC: right-sided colorectal cancer
TTD: time to treatment discontinuation
TCR: Taiwan Cancer Registry
